# Comparative proteomics and metabolomics reveals distinct host protein quality control and metabolic signatures during recombinant IL1-His and IL15-His expression in *Nicotiana benthamiana*

**DOI:** 10.1371/journal.pone.0353563

**Published:** 2026-07-20

**Authors:** Alina Savinova, Theerakarn Srisangsung, Pipob Suwanchaikasem, Waranyoo Phoolcharoen

**Affiliations:** 1 Department of Pharmacognosy and Pharmaceutical Botany, Faculty of Pharmaceutical Sciences, Chulalongkorn University, Bangkok, Thailand; 2 Center of Excellence in Plant-produced Pharmaceuticals, Chulalongkorn University, Bangkok Thailand; 3 Baiya Phytopharm, Bangkok, Thailand; Stony Brook University, UNITED STATES OF AMERICA

## Abstract

Agrobacterium-mediated transient expression in *Nicotiana benthamiana* is widely used for recombinant biopharmaceutical production. To investigate the host plant response upon human cytokine production with contrast accumulation, we expressed codon optimized human IL1 beta, serving as a high expression benchmark with a yield of approximately 80 μg/g leaf fresh weight, and IL15 with undetected signal on western blot, representing protein with low level of accumulation, using the geminiviral vector system. A combined proteomics and metabolomics technique was applied to elucidate underlying cellular mechanisms. Quantitative proteomics revealed that IL1-His was robustly detected (9 unique peptides, 83% coverage), whereas no IL15-His–derived peptides were identified. Based on pathway analysis, the expression of IL15-His induced chaperone expression, with downregulation of photosynthetic and primary metabolism pathways. Additionally, metabolomic pathway analysis revealed that IL1-His preferentially drives the branched chain amino acid biosynthesis, but IL15-His shifts metabolism towards phenylpropanoid biosynthesis routes. These findings underscore that the characteristics of the target protein and its interaction with the host's physiology could influence the yield of the recombinant protein production in plants.

## Introduction

The production of recombinant and therapeutic proteins in plants (molecular farming) has been established as a promising alternative to conventional microbial and mammalian expression systems. Plant-based expression platforms are recognized for their cost-effectiveness, scalability, and biosafety [1].

Among the plant hosts, *Nicotiana benthamiana* has become the “model plant” for transient agroinfiltration due to its fast growth, significant leaf biomass, and sensitivity to *Agrobacterium* infection [2]. Unlike stable transgenic approaches, agroinfiltration does not integrate foreign DNA into the plant genome; bacterial vectors introduce transgenes into leaf cells, driving high-level but temporary expression. This method is both rapid and high yielding because recombinant proteins can accumulate significantly within 4−7 days post-infiltration [3]. A wide array of recombinant biopharmaceuticals was successfully produced in plants. Early studies demonstrated production of human granulocyte-macrophage colony-stimulating factor (rhGM-CSF) in rice seeds [4], and biologically active human Interleukin-2 produced in potato plants [5]. Subsequent development has demonstrated that plants can support the efficient production of clinically relevant therapeutic proteins, including human interferon alpha 2b [6], proinsulin [7] and antibodies [8]. These and other case studies demonstrate the potential for plants to serve as bio factories for cytokines and other complex human therapeutic proteins, providing economic and safety advantages [9].

While most molecular farming research focused on improving the yield of the target recombinant protein, there is a growing recognition that understanding the mechanisms underlying the host plant’s response is crucial for optimizing production. Proteomics has emerged as a conventional system and a valuable tool in biotechnology. In the context of recombinant protein production, proteomics can provide direct measurements of the proteins – their abundances, modifications, and subcellular localization [10]. Modern proteomic methods such as data-dependent acquisition (DDA) and data-independent acquisition (DIA), along with precise quantification algorithms, including label-free quantification (LFQ) and tandem mass tag labeling (TMT), revealed how the host organism responds to the burden of heterologous protein expression. These approaches have enabled researchers to identify critical factors affecting protein production efficiency, such as the impact of bacterial vector cultivation conditions on recombinant target protein yield [11] and the role of host proteases in the recombinant protein degradation [12]. Multi-omics investigations of *N. benthamiana* leaves upon agroinfiltration have shown how plants initiate the proteolytic activity toward *Agrobacterium* vector infiltration. They found that expression of the P19 viral silencing suppressor showed little effect on the proteome, extracellular proteome and secretome [13]. In addition, targeted proteomics studies have provided insight into the physiological burden associated with heterologous protein accumulation. For example, during the expression of complex secretory proteins, proteomic profiling revealed an early and transient increase in unfolded protein response (UPR)-related machinery, suggesting that ER protein quality control responses can be activated before late-stage defense responses and leaf necrosis typically observed at peak protein accumulation [14]. Together, these studies demonstrated the power of proteomics to provide valuable insight for the refinement of expression systems for better productivity.

Metabolomic serves as a vital complementary analysis to proteomics for understanding the physiological state of the plants. Unlike the proteins, which indicate the potential cellular machinery, metabolites are the end products of the gene expression, protein activity, offering the quantitative readout of the plant’s physiological status [15]. This approach is crucial for obtaining a broader picture about cellular responses to environmental stresses or infection [16]. Several studies indicate great metabolomic reprograming and induction of the production of signal molecules upon agroinfiltration [11,17]. Identifying these dynamic changes can reveal crucial bottlenecks that limit recombinant protein production.

Cytokines are small signaling proteins that regulate immune responses, inflammation, and cellular communication. Human IL1 and IL15 are critically important immunoregulatory cytokines, whose recombinant forms are used as standards, adjuvants, and therapy candidates [18,19]. While both are essential for immune system functioning, they differ significantly in their mechanism of action: IL1 beta plays the central role in the induction of inflammatory responses, making it both a therapeutic agent and an immunomodulator in cancer therapy [20,21]. Meanwhile, IL15 maintains the lymphocyte populations and enhances the proliferation of NK cells and CD8 + T cells, making it useful in immunotherapy and vaccine adjuvant applications [22,23]. The production of these cytokines in plant expressing systems could offer substantial advantages over the conventional expression system, including reduced cost, enhanced safety due to the absence of human pathogens, and the ability to perform the post-translational modifications that are often critical for biological activity [24]

Despite the therapeutic importance, these cytokines are difficult to produce in a heterologous host, partly because of their complex folding conformation and hydrophobic cores. IL15 has a compact four-helix bundle with several hydrophobic surfaces that must be packed very precisely to bind its receptor and remain stable; molecular dynamics simulations show that small changes in these hydrophobic regions can strongly affect its stability [25]. IL1 beta has a *β-*trefoil fold, where three subdomains are packed around tightly packed hydrophobic core. Folding simulations indicate that probability of existence of partially folded molecules before the native state is reached [26]. In our transient expression system, IL1-His showed detectable accumulation, whereas IL15-His was not detected by the analytical methods used in this study.

Based on this contrast, we hypothesized that high and low cytokine accumulation in *N. benthamiana* could be associated with distinct host proteomic and metabolic response signatures. Therefore, the goal of this study was to compare proteome and metabolome changes in transiently agroinfiltrated *N. benthamiana* leaves expressing IL1-His and IL15-His using the same geminiviral vector system. Rather than serving only as a descriptive analysis, these proteomic and metabolomic data were used to identify candidate host processes that may be associated with recombinant cytokine accumulation. Such information can guide future optimization strategies, including protein engineering, co-expression of folding assistants or protease inhibitors, and modulation of host metabolic or stress-response pathways to improve the consistency and yield of therapeutic cytokine production in plant systems.

## Materials and methods

### Vector construction for human interleukin expression

For expression of human interleukin 1 (IL1) and human interleukin 15 (IL15) in *Nicotiana benthamiana,* coding nucleotide sequences of IL1 (PDB: 3O4O_A) and IL15 (PDB: 2XQB_A) were codon optimized for expression. They were *de novo* synthesized using GeneArt gene synthesis (ThermoFisher Scientific, USA). The sequences were engineered to include a signal peptide in the N-terminal and the tag coding eight consecutive histidine residues (8xHis) in the C-terminal, followed by the SEKDEL sequence for protein retention within the endoplasmic reticulum [27]. Each synthetic gene was flanked by *XbaI* and *SacI* restriction sites for directional cloning and was subsequently ligated into geminiviral pBYR2eK (pBY), using the standard ligation-restriction method [28]. The expression vectors were transformed into *Agrobacterium tumefaciens* GV3101 (Cat. no. CC-207-A, Goldbio,USA) using electroporation. The strain has a C58 chromosomal background with rifampicin resistance and carries the disarmed Ti plasmid pMP90 (pTiC58ΔT-DNA), which confers gentamicin resistance. Kanamycin was used for selection of binary expression vector.

### Agroinfiltration and production of recombinant proteins in *Nicotiana benthamiana*

Transformed *A. tumefaciens* were cultured 16 h at 28 °C in 10 g/L tryptone, 5 g/L yeast extract, 10g/L NaCl media with 50 µg/ml rifampicin, 50 µg/ml gentamycin, and 50 µg/ml kanamycin. The next day, the bacterial suspension was centrifuged at 7,000 × g for 5 min and resuspended in 1X infiltration buffer (10mM magnesium sulfate at pH 5.5 and 10 mM 2-N-morpholino-ethanesulfonic acid (MES)) to a final OD600 of 0.2. Using the 1 ml needleless syringe, the bacterial suspension was carefully infiltrated into the stomatal side of the three to four leaves of 3–4 week old plants *N. benthamiana,* applying gentle pressure. After infiltration, the plants were maintained in a controlled environment with a 16 h light and 8 h dark photoperiod at a temperature of 28 °C. The infiltrated leaves were harvested for analysis 4 days post-infiltration (dpi) [29].

### Protein extraction

For protein extraction, infiltrated leaves were collected at 4 dpi in the 1.5 ml tubes. Lysis buffer (20 mM Tris-HCl pH 7.4, 50 mM NaCl, 5 mM imidazole) was added at ratio 100 μl buffer per 100 mg of leaf. Homogenization was performed with stainless steel beads (2 mm in diameter) in a mixer mill MM 400 (Retsch, Germany) at 30 Hz for two cycles of 3 min each at room temperature [30]. The crude extract was clarified at 14,000 g for 10 min at 4 °C. Three volumes of ice-cold methanol were added to precipitate the total proteins from clarified supernatant, following the incubation at −20 °C for 5 min. The precipitated proteins were separated by centrifugation at 14,000 g for 10 min at 4 °C, and the pellet was resuspended in 50 mM ammonium bicarbonate buffer, pH 7.8. Total protein concentration was measured by Bradford assay (Bio-Rad, USA), and all leaf extract samples were normalized to a final concentration of 0.7 mg/ml for downstream analysis.

### SDS gel electrophoresis and Western blot

For the detection of IL1-His and IL15-His in the leaf extracts using SDS-PAGE and Western blot, crude leaf extracts were mixed with 6x non-reducing loading buffer (125 mM Tris-HCl, pH 6.8, 12% SDS, 10% glycerol, and 0.001% of bromophenol blue). A total of 10 µg of protein per sample, along with a crude extract of FGF2-His protein (produced in-house), was loaded onto 8% or 4−15% gradient polyacrylamide gels and separated according to the Laemmli method using Mini-PROTEAN tetra cell (Bio-Rad, USA) [31]. Following the electrophoresis, one gel was stained with Coomassie to visualize the total protein profile. Proteins were transferred into the nitrocellulose membrane for immunodetection using Mini Transblot Cell (Bio-Rad, USA). Following the transfer, the membrane was blocked with 5% skim milk in PBS + 0.01% Tween for 1 h. The membrane was incubated with Mouse anti-His-Tag HRP antibody (Cat. no. 4603−05, Southern Biotech, USA) in dilution 1:5000 in 3% skim milk for 1 h [32]. Protein bands were visualized using an enhanced chemiluminescent ECL substrate kit (Cat. no. ab133409, Abcam, USA). The signal from the Coomassie Blue-stained gel and the chemiluminescent blot was captured with an ImageQuant LAS 500 (GE Healthcare, Germany). The images were analyzed using ImageLab Software (Bio-Rad, USA). Raw file of SDS-PAGE and Western blot analysis is provided in S1 Fig.

### Proteomics analysis by LC-MS

For proteomic profiling, 20 µg of each leaf protein extract was prepared for mass-spectrometry analysis. The samples were first reduced with 10 mM of dithiothreitol (DTT) at 65 °C for 30 min, then alkylated with 25 mM iodoacetamide (IAA), and then protein samples were digested overnight using trypsin. The digestion was quenched by adding formic acid, and any insoluble material was removed by centrifugation [33]. Proteomic measurements were performed in three independent replicates. The digested peptides were analyzed using an Agilent LC-QTOF 6545XT mass spectrometry (MS) system (Agilent, USA). Chromatographic separation was performed on an Agilent Peptide Map column (2.1 × 150 mm, 2.7 µm), using the binary solvent system, consisting of water with 0.1% formic acid and acetonitrile with 0.1% formic acid. The peptide elution was achieved with a linear gradient of 0–90% B over 75 min. The instrument was set to acquire MS1 scans over the mass range 40–1700 m/z, followed by data dependent MS2 fragmentation scans of selected precursors over the 25–1000 m/z range. MS parameters can be found in S1 Table.

### Proteomics data analysis

The tandem mass spectra were searched against the concatenated database constructed from the reference proteomes of *Nicotiana benthamiana* (TaxonID: 4100), *Nicotiana tabacum* (TaxonID: 4097), *Nicotiana attenuata* (TaxonID: 49451), *Nicotiana sylvestris* (TaxonID: 4096), and *Agrobacterium tumefaciens* (TaxonID: 176299) downloaded from Uniprot database. The FASTA sequences of IL-1 and IL-15 were also added, along with a built-in list of common laboratory contaminants. The mass tolerance for the first peptide search was defined as 20 ppm and refined to 10 ppm for the main search. For quantification, the MaxLFQ algorithm was used [34]. Both peptide-spectrum matches, and protein identification were filtered using the target-decoy approach to achieve the false discovery rate (FDR) of 1%. The final output “ProteinGroups.txt”, “evidence.txt”, “msms.txt” raw files were used for quality control assessment, statistical analysis, and biological interpretation (S2-S3 Fig).

Proteomic analysis and visualization were performed in Perseus (version 1.6.14.0) [35]. The initial protein list was filtered to remove identifications from the reverse database, known contaminants, and proteins identified only by modified peptides. For quantitative analysis, LFQ intensities were first log2 transformed, and filtered according to the criteria (100% valid numbers at least in 1 group). Missing values were imputed from a normal distribution using a formula of width = 0.3 SD and downshift = 1.8 SD as a default setting in the software. Exploratory data analysis included principal component analysis (PCA), Pearson correlation analysis and LFQ intensity distribution (S4 Fig). To identify the proteins with significant changes, a two-sided Student’s T-test was applied for each protein with multiple comparison correction, using the Benjamini-Hochberg false discovery rate (FDR) method, with significance threshold set at FDR < 0.05 and S = 0.1.

Gene Ontology analysis was performed using Fisher's exact test with a one-tailed alternative hypothesis, where a 2 × 2 contingency table compared the number of input genes annotated to each GO term versus background gene annotations. P-values were corrected for multiple comparisons using the Benjamini-Hochberg FDR method, with a significance threshold set at FDR < 0.05. Fold enrichment was calculated as the ratio of observed to expected gene counts in each GO term, where expected genes = (Total input genes × Total background genes in GO term) / Total background genes.

### Metabolomic analysis by LC-MS

For metabolomic profiling 100 mg of fresh leaf was homogenized with 100 μl of lysis buffer (20 mM Tris-HCl pH 7.4, 50 mM NaCl, 5 mM imidazole) in a mixer mill MM 400 (Retsch, Germany) at 30 Hz for two cycles of 3 min each at room temperature. After centrifugation at 14,000 g for 10 min, three volumes of ice-cold methanol with 50 ng/ml of sulfadimethoxine (Cas no. 122-11-2) were added. Precipitated proteins were removed by second centrifugation at 14,000g for 5 min, and supernatant was collected. A 10 μl of methanolic extract was injected onto the column Agilent InfinityLab Poroshell (120 EC-C18, 2.1 x 100 mm, 2.7 μm). Metabolites were separated using the binary solvent system, consisting of water with 0.1% formic acid (solvent A) and the acetonitrile with 0.1% formic acid (solvent B). Elution was performed with linear gradient 0–90% B over 20 min. The mass spectrometer was operated in positive and negative mode to acquire MS1 scans over the mass range 40–1700 m/z, followed by data dependent MS2 fragmentation scans of selected precursors over the 25–1000 m/z range. Other MS parameters can be found in the S2 Table.

To monitor analytical performance, a quality control (QC) sample was prepared by pooling all samples together. The pooled QC was injected at the beginning of the run and every 5 samples throughout the batch to assess the chromatographic and mass spectrometer stability. In addition, QC dilution series (0, 1, 10, 20, 50 and 80% dilutions), prepared from the pooled QC sample in corresponding extraction solvent with internal standard, was analyzed to evaluate the signal linearity. Only features exhibiting the monotonic response across the dilution series were retained for downstream analysis.

### Metabolomics statistical analysis and bioinformatics

Metabolomic analysis was performed using MS-DIAL [36] for peak detection, deconvolution, alignment, and metabolite identification, and Metaboanalyst 6.0 [37] for downstream statistical analysis. Untargeted metabolite identification was based on MS/MS matching against RIKEN tandem mass spectral database (ReSpect) for phytochemicals [38], the Fiehn/Vaniya Natural Products Library [39], the Global Natural Products Library [40], and an in-house ESI(+)-MS/MS library acquired from authentic standards (version 17). Peak intensities were normalized using the LOWESS method and internal standard (sulfadimethoxine). After normalization the features were retained only if they satisfied following quality criteria: Pearson correlation coefficient with dilution QC samples ≥ 0.70, coefficient of variation in pooled QC injections ≤ 30%, mass error between −20 and +20 ppm, and total identification score ≥ 0.7. The resulting set of metabolites was exported to Metaboanalyst 6.0 for statistical analysis and visualization.

For each pairwise comparison between experimental groups, univariate testing was performed, using unpaired two-tailed Student’s T-test, and log2-fold changes were calculated from the normalized intensities. The metabolites were first visualized as clustered heatmap. Prior to visualization, data was log10-transformed and auto-scaled (mean-centered and divided by the standard deviation). Hierarchical clustering of both metabolites and samples was performed on the Z-scored intensities using Euclidean distance and Ward’s linkage. Volcano plots were generated with a raw P-value cutoff of 0.05 and an absolute log2 fold change ≥ 1 (corresponding to a fold change ≥ 2). Metabolites meeting both criteria were considered significantly altered.

Significantly up- and down-regulated metabolites were subsequently annotated with KEGG compound identifiers and subjected to pathway analysis using the Pathway Analysis module of MetaboAnalyst 6.0. Pathway over-representation was evaluated using Fisher’s exact test against KEGG metabolic pathways, while pathway topology was assessed using relative betweenness centrality. *Arabidopsis thaliana* was selected as a model organism for running pathway impact analysis.

## Results

### Transient expression of IL1-His and IL15-His in *Nicotiana benthamiana*

**Fig 1:**
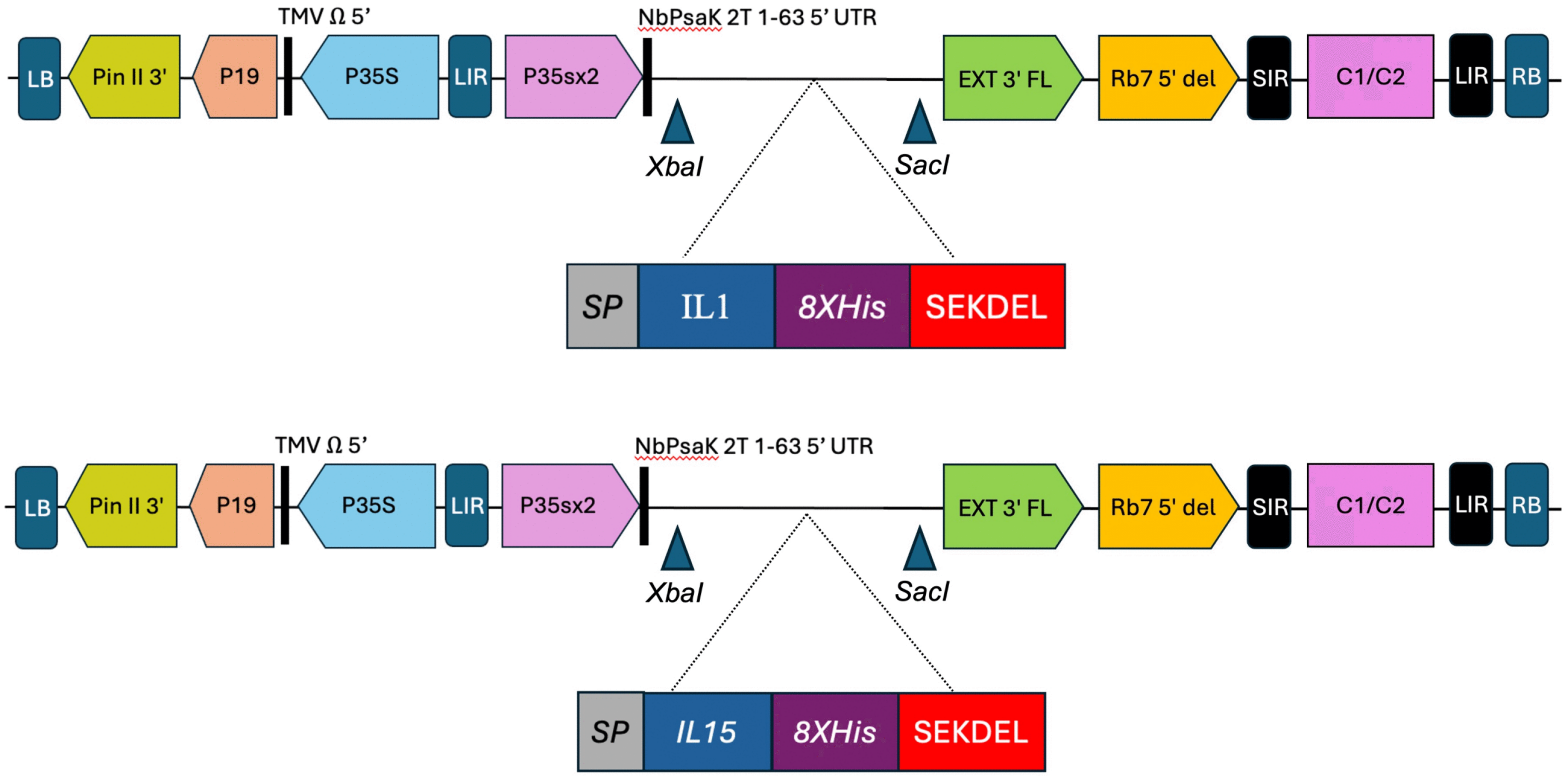
Vector construction for IL1-His and IL15-His expression. Codon-optimized sequences were cloned into the pBY vector. LB/RB –– left and right T-DNA border, Pin II 3’ –– potato protease inhibitor II border element, P19 –– tomato bushy stunt virus gene silencing suppressor, TMV 5’UTR 5’ –– untranslated region of Tobacco Mosaic Virus Ω, NbPsaK2T 1-63 5’UTR –– 5’ untranslated region derived from *N. benthamiana* photosystem K subunit. Ext 3’ –– tobacco extension 3’ element, Rb7’del –– tobacco Rb7 matrix attachment element. SIR/LIR –– short and long intragenic region, C1/C2 ––replication-associated genes [42–44].

To compare the expression of human interleukin IL1-His and IL15-His in plants, their codon–optimized coding sequences, flanked with N-terminal signal peptide and 8xHis-tag followed by SEKDEL peptide for ER retention at the C-terminal, were cloned into geminiviral expression vector pBYR2eK (pBY) [41] (Fig 1). These expression vectors and an empty pBY vector as a control were transformed into *Agrobacterium tumefaciens GV3101.* Cultures of transformed *A. tumefaciens* were used for infiltration of *N. benthamiana* leaves.

**Fig 2:**
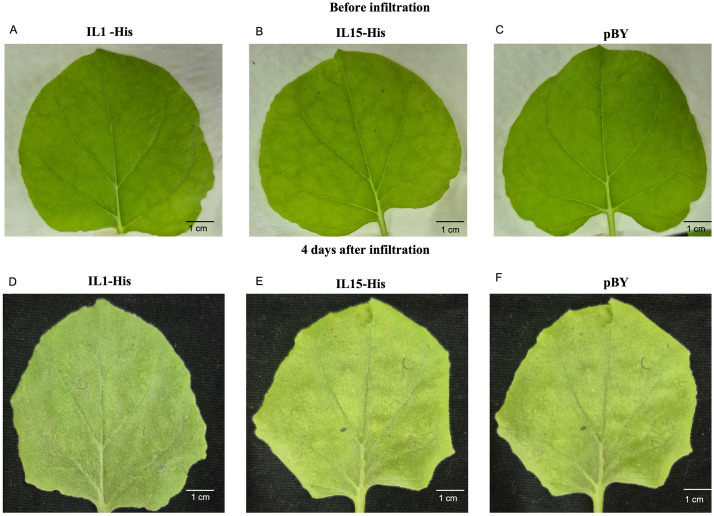
Necrotic symptom development. Leaves expressing IL1-His (A and D), IL15-His (B and E), and pBY control (C and F) before infiltration (A, B, and C) and 4 days after infiltration (D, E, and F).

Before infiltration, all plants appeared healthy across all groups (Fig 2A, 2B and 2C). After 4 days of post-infiltration (dpi), leaves demonstrated significant necrosis in the infiltrated zones. Plants infiltrated with IL1-His showed extensively affected tissues at the infiltrated areas (Fig 2D). Similarly, IL15-His infiltrated plants exhibited comparable necrotic symptoms, displaying pronounced yellow discoloration (Fig 2E). Plants infiltrated with empty pBY control vector also developed significant necrosis, visibly indistinguishable from those observed in the protein-expressing plants (Fig 2F). Individual leaf analysis revealed that tissue damage between IL1-His, IL15-His, and pBY was similar, suggesting that the observed necrosis was associated with agroinfiltration and geminiviral vector replication.

**Fig 3:**
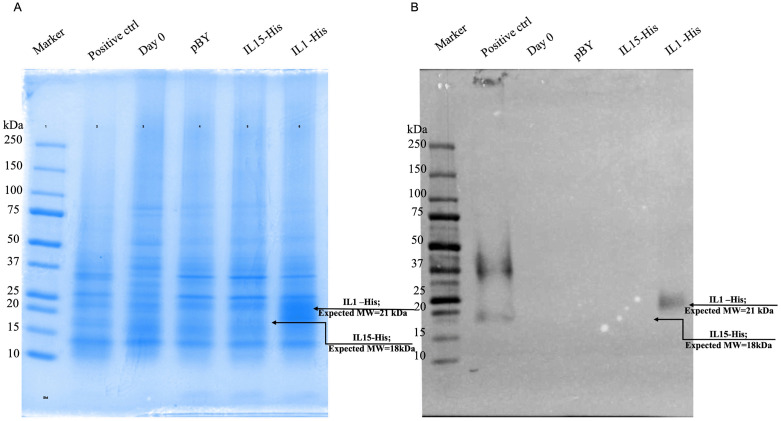
Non-reducing SDS-PAGE (A) and Western blot (B) analysis of total soluble proteins extracted from agroinfiltrated *N. benthamiana* leaves. Expected molecular weight of IL1-His is 21kDa and IL15-His is 18kDa. Lanes labeled: Marker – Precision Dual Color Protein Ladder (Cat. No. 1610373EDU, Bio-Rad, USA); Positive control –FGF2-His protein; Day 0 – non-infiltrated leaf sample; pBY – empty vector.

To analyze protein expressions, total soluble proteins were extracted from leaf samples collected after 4 dpi. Distinct bands corresponding to the IL1-His and IL15-His were not clearly visible by Coomassie staining (Fig 3A). Western blot analysis with anti-His HRP-conjugated antibody revealed a clear band at the expected molecular weight, approximately 21 kDa, for IL1-His in crude leaf extracts (Fig 3B). In comparison, no band for IL15-His (expected at 18 kDa) was detected under the same conditions, indicating that accumulation of IL15-His was below the limit of detection for this method. The in-house-produced FGF2-His protein was used as a positive control for the anti-His Western blot assay. These results demonstrate a successful expression of IL1-His. Based on the semi-quantitative densiometric analysis of SDS-PAGE and Western blot, using the known loading amount and amount of leaf material used for extraction, IL1-His accumulation was estimated to be approximately 80 μg protein/g leaf fresh weight. Despite the similar necrotic response, the difference in accumulation shown in the western blot indicates plant respond differently upon production of the recombinant interleukins in *N. benthamiana*, and different pathway could be induced, which could be elucidated through subsequent proteomic analysis.

### Global proteome analysis reveals specific expression signatures

LC-MS proteomic profiling of total soluble fractions from infiltrated leaf samples was performed to investigate the molecular basis underlying the differential production yields of IL1-His and IL15-His in *N. benthamiana*. In total, 593 peptides and 171 protein groups were identified. Following strict filtering and imputation, 155 high-confidence proteins were retained for downstream analysis (S3 Table).

**Fig 4:**
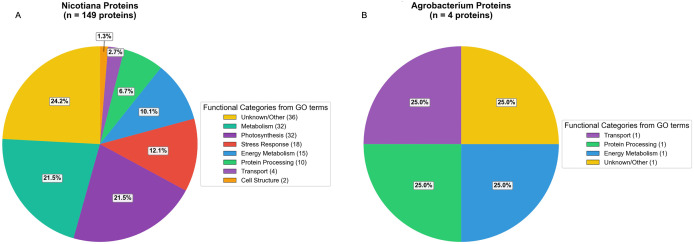
Taxonomic distribution and functional categorization of the identified proteins during the transient expression in *N. benthamiana* leaves. Complete dataset of 155 high-confidence proteins between the *Nicotiana* (149 proteins) (A) and *Agrobacterium* (4 proteins) species (B). Pie charts show gene ontology-based functional categories: Yellow = Unknown/Other; Purple = Stress Response; Blue = Energy Metabolism; Green = Protein Processing; Red = Metabolism; Light purple = Transport; Orange = Cell Structure.

To investigate the distribution of host and bacterial proteins in our dataset and understand general cellular processes actively engaged during transient protein production, taxonomic annotation, and functional categorization of the 155 quantified proteins were performed. Host plant proteins are prevalent in the dataset (Fig 4A). There were 149 *Nicotiana* proteins (96.1%) distributed across functional categories of photosynthesis (21.5%), general metabolism (21.5%), stress response (12.1%), energy metabolism (10.1%), transport (2.7%), and other/unknown functions (24.2%). Only 4 proteins (3.9%) were identified as *Agrobacterium tumefaciens* proteins, categorized into protein processing (25.0%), transport (25.0%), energy metabolism (25.0%), and unknown function proteins (25.0%) as shown in Fig 4B. The minimal detection of bacterial proteins demonstrates the authentic plant physiological responses to transient protein expression.

**Fig 5:**
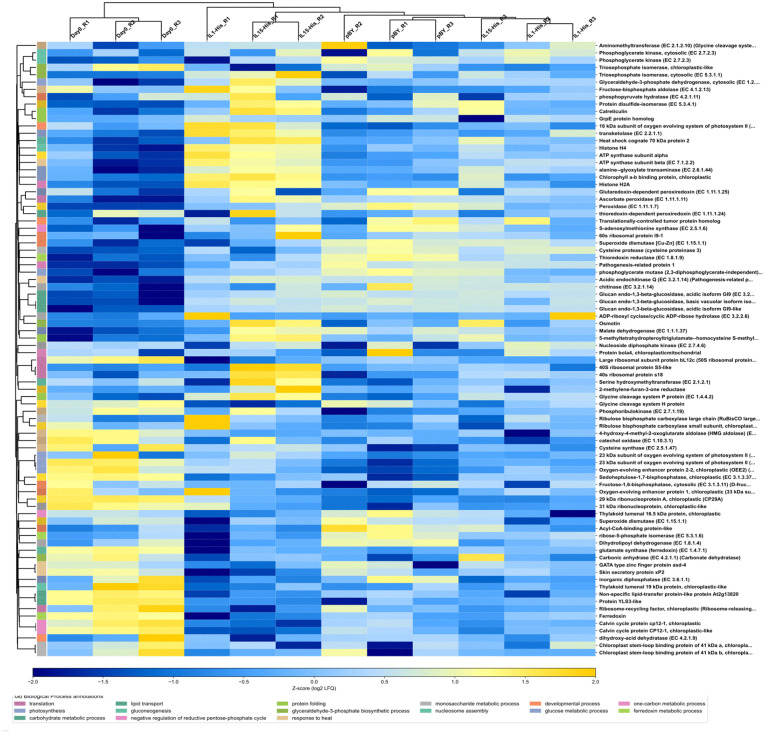
Hierarchical clustering. Columns represent individual samples, and rows represent proteins. Rows are clustered by Gene Ontology Biological Processes (GO BP) similarity, using the weighted Jaccard distance. Columns are clustered by average-linkage hierarchical clustering on Euclidean distances computed from log2-transformed, row-wise Z-scored intensities The color scale indicates the row-wise Z-score, with yellow representing high relative abundance and blue representing low relative abundance. Each column represents an independent replicate (n = 3 per condition). The colour strip denoted the dominant GO BP term per protein.

To investigate plant response during the expression of recombinant IL1-His and IL15-His, we performed hierarchical clustering of the identified proteins and visualised the data as the heatmap of Z-score log2-transformed intensities. Only proteins which were annotated with Gene Ontology (GO) Biological Process terms retained for clustering. The hierarchical clustering revealed distinct protein expression signatures segregated by expressed constructs despite some variation among biological replicates (Fig 5). Healthy non-infiltrated plants (Day 0) are characterized by a cluster of upregulated proteins (yellow), associated with photosynthetic machinery, including proteins of ribulose bisphosphate carboxylase, ferredoxin, thylakoid proteins, and Calvin cycle protein (CP12). Upon the agroinfiltration, proteome remodelling patterns emerged across the groups. Plants infiltrated with pBY empty vector revealed the baseline agroinfiltration response, showing mild upregulation of defence proteins, including pathogen-related proteins, chitinase, and β-glucosidase. By comparison with pBY—heterologous interleukin expression, distinct proteome changes were induced. IL15-His expression triggered the most proteome remodelling, characterized by activation of stress response proteins (heat shock or chaperone proteins), accompanied by substantial suppression (blue) of photosynthetic and Calvin cycle proteins (ribulose bisphosphate carboxylase and triosephosphate isomerase). By contrast, IL1-His infiltrated plants exhibit moderate proteome adjustment, maintaining relatively stable photosynthetic and defence protein levels.

These differences in proteome signatures suggest that the expression of recombinant IL1-His and IL15-His affects host plant physiology in different ways. IL15-His transient expression could be associated with a stronger stress response, which may contribute to its low accumulation, while IL1-His transient expression appeared to be better tolerated at metabolic level.

### Quantitative assessment of plant proteome remodeling upon recombinant interleukin production

**Fig 6:**
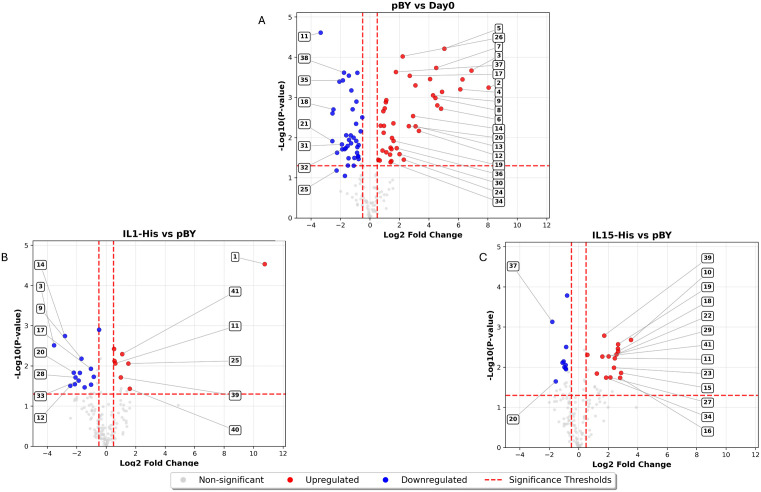
Volcano plots of proteins altered by IL1-His, IL15-His expression, and pBY empty vector. Each panel shows a pairwise comparison: (A) Day 0 versus empty vector pBY agroinfiltration, (B) IL1-His expression versus empty vector pBY, and (C) IL15-His expression versus empty vector pBY. X-axis is a log2 fold change, and Y-axis is a -log10(p-value). Red points are significantly upregulated, blue points are downregulated considerably, and gray points are non-significant proteins. Dashed lines indicate significance thresholds (p < 0.05 and |Log2FC| > 1.0). Number in the boxes refers to protein number, listed in Table 1.

Differential expression analysis was performed using univariate testing with Benjamini-Hochberg correction for multiple comparisons across the three biological replicates per condition to translate the global proteomic shifts into functional insights and identify the host processes associated with the low protein yield of IL15-His. The analysis identified 78, 26, and 30 significantly differentially expressed proteins in 3 comparisons between pBY vs Day0, IL1-His vs pBY, and IL15-His vs pBY samples, respectively **(**Fig 6). Table 1 and S4 Table show the proteins that changed according to the comparison between all conditions.

**Table 1:** Significantly different abundant proteins highlighted in the volcano plot.

No.	Protein name	Protein ID	log2(FC)		
			pBY vs Day0	IL1-His vs pBY	IL15-His vs pBY
1	Interleukin-1 beta (IL-1 beta) (Catabolin)	P01584	n.s.	10.750	n.s.
2	Glucan endo-1,3-beta-glucosidase, acidic isoform GI9-like	A0A1U7WQQ4	8.048	n.s.	n.s.
3	Peroxidase (EC 1.11.1.7)	A0A1J6JRZ7	6.883	−3.540	n.s.
4	Glucan endo-1,3-beta-glucosidase, acidic isoform GI9 (EC 3.2.1.39) ((1- > 3)-beta-glucan endohydrolase) ((1- > 3)-beta-glucanase) (Beta-1,3-endoglucanase) (PR-2B) (PR-36)	P23547	6.131	n.s.	n.s.
5	Acidic endochitinase Q (EC 3.2.1.14) (Pathogenesis-related protein Q) (PR-Q)	P17514	5.046	n.s.	n.s.
6	Superoxide dismutase [Cu-Zn] (EC 1.15.1.1)	A0A1U7XJM1	4.828	n.s.	n.s.
7	Glucan endo-1,3-beta-glucosidase, basic vacuolar isoform isoform X2	A0A1U7VEP8	4.491	n.s.	n.s.
8	chitinase (EC 3.2.1.14)	Q9FEW1	4.443	n.s.	n.s.
9	Pathogenesis-related protein r major form	A0A314KT76	4.275	−1.677	n.s.
10	Chlorophyll a-b binding protein, chloroplastic	A0A1J6K2T4	n.s.	n.s.	3.546
11	Carbonic anhydrase (EC 4.2.1.1) (Carbonate dehydratase)	A4D0J8	−3.353	0.610	2.437
12	Trehalose utilization-related protein	A9CF14	3.314	−2.436	n.s.
13	Profilin	A0A1U7WGL4	3.094	n.s.	n.s.
14	Dienelactone hydrolase domain-containing protein	A0A1J6IXF2	2.927	−2.806	n.s.
15	Chaperone protein DnaK (HSP70) (Heat shock 70 kDa protein) (Heat shock protein 70)	A0A176X794	n.s.	n.s.	2.865
16	40s ribosomal protein s18	A0A1J6IPD7	n.s.	n.s.	2.806
17	Thioredoxin reductase (EC 1.8.1.9)	A0A1U7W0E9	2.697	−1.039	n.s.
18	NAD(P)H dehydrogenase (quinone) (EC 1.6.5.2)	A0A1U7X681	−2.479	n.s.	2.668
19	Histone H2A	A0A1J6JIC4	1.597	n.s.	2.664
20	Calmodulin (Calmodulin NtCaM2)	Q76MF4	2.635	−2.201	−1.565
21	Carbonic anhydrase (EC 4.2.1.1) (Carbonate dehydratase)	A0A314KZ99	−2.559	n.s.	n.s.
22	Osmotin	P14170	n.s.	n.s.	2.558
23	Calreticulin	A0A1S4C3F4	n.s.	n.s.	2.389
24	chitinase (EC 3.2.1.14)	A0A1S3YRD6	2.292	n.s.	n.s.
25	Histone H3.3-like	A0A1U7XR19	−2.264	1.501	n.s.
26	Cysteine protease (cysteine proteinase 3)	Q9LRI2	2.216	n.s.	n.s.
27	Histone H4	A0A9R6	n.s.	n.s.	2.146
28	Thylakoid lumenal 16.5 kDa protein, chloroplastic	A0A1U7UVW6	n.s.	−2.086	n.s.
29	ATP synthase subunit alpha	A0A140G1P7	n.s.	n.s.	2.020
30	Peroxidase (EC 1.11.1.7)	A0A1U7VMV2	2.007	n.s.	n.s.
31	Phosphoribulokinase (EC 2.7.1.19)	A0A1S3X711	−1.909	n.s.	n.s.
32	Protein YLS3-like	A0A1U7VFH1	−1.882	n.s.	n.s.
33	Superoxide dismutase (EC 1.15.1.1)	A0A1S3XPG2	n.s.	−1.869	n.s.
34	monodehydroascorbate reductase (NADH) (EC 1.6.5.4)	A0A1S3YUT4	1.399	n.s.	1.859
35	glutamate synthase (ferredoxin) (EC 1.4.7.1)	A0A1S3Z1F3	−1.842	n.s.	n.s.
36	Protein disulfide-isomerase (EC 5.3.4.1)	A0A9E7VCB0	1.815	n.s.	n.s.
37	LysM domain-containing GPI-anchored protein 2	A0A1U7VCZ8	1.754	n.s.	−1.804
38	glutamate synthase (ferredoxin) (EC 1.4.7.1)	A0A1U7Y601	−1.763	n.s.	n.s.
39	alanine--glyoxylate transaminase (EC 2.6.1.44)	A0A1U7WMP7	n.s.	0.989	1.722
40	ATP synthase subunit beta (EC 7.1.2.2)	A0A140G1S2	n.s.	1.599	n.s.
41	Glycine cleavage system P protein (EC 1.4.4.2)	A0A1U7YE93	n.s.	1.085	1.594

The plants infiltrated with pBY demonstrated dramatic cellular reprogramming, with 78 significant proteins (43 upregulated and 35 downregulated) (Fig 6A). The upregulated proteins (right side of the volcano plot) were dominated by markers of plant defense, including glucan endo 1,3-*β*-glucosidase, acidic endochitinase Q and pathogen related proteins [45,46], the downregulated proteins (left side of the volcano plot) were strongly associated with photosynthesis and primary metabolism, including oxygen-evolving enhancer proteins; ferredoxin and carbonic anhydrase [47–49].

In the comparison of IL1-His expressing leaves with the empty pBY control the proteomic changes were less dramatic, with only 26 significant proteins identified (8 upregulated and 18 downregulated) (Fig 6B). Upregulated proteins consisted mainly of proteins related to glycine metabolic process, including glycine cleavage system P protein and photosynthesis proteins, including phosphoribulokinase, oxygen-evolving enhancer protein, and photosystem I assembly protein [49,50]. Downregulated proteins were phosphoglycerate mutase, associated with anaerobic glycolysis [51], and peroxidase and thioredoxin reductase, involved in superoxide removal [52,53].

In the comparison of IL15-His with empty pBY control, a similar number of significantly changed proteins were observed, with 30 significant proteins (19 upregulated and 11 downregulated) (Fig 6C). The IL15-His protein was not identified in the proteome analysis. The upregulated proteins in IL15-His condition were similar to IL1-His condition, comprising proteins involved in glycine metabolic process and photosynthesis, including alanine-glyoxylate transaminase [39], chlorophyll a-b binding protein, and 23 kDa subunit of oxygen-evolving system of photosystem II [50,54]. However, unique proteins for this condition, linked to stress response and heterochromatin organization, were detected, including heat shock cognate 70 kDa protein 2, osmotin, calreticulin, and histone H2A. The most significant downregulated proteins were associated with metabolic and regulatory processes [42].

**Fig 7:**
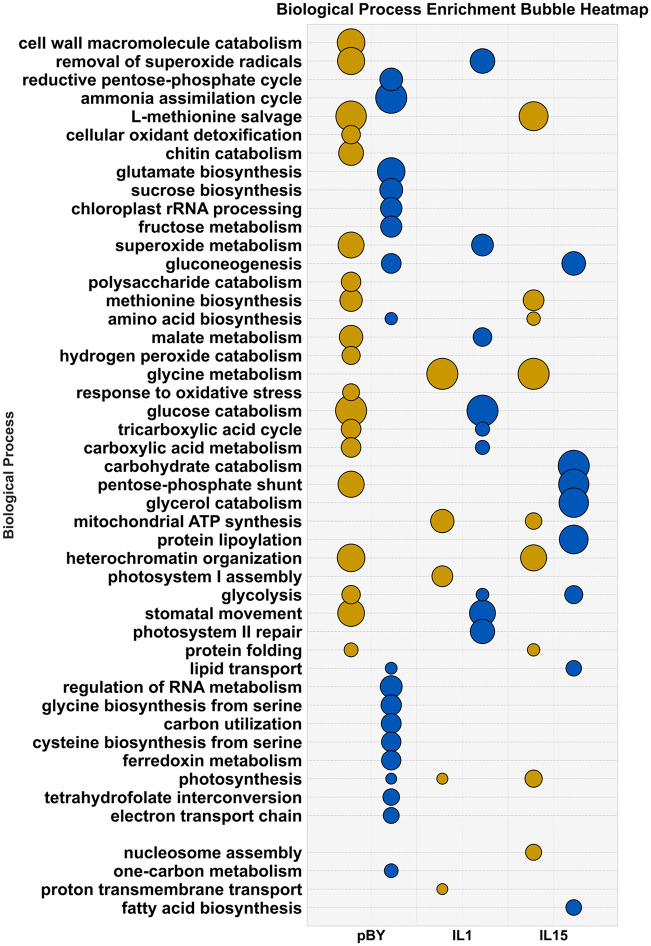
Gene Ontology biological process enrichment of differentially abundant proteins in infiltrated leaves, summarized as a bubble heatmap. Columns represent three pairwise comparisons: pBY vs Day0, IL1-His vs pBY, and IL15-His vs pBY. Rows correspond to significantly enriched GO biological processes. Each bubble indicates an enriched process for either upregulated (yellow) or downregulated (blue) proteins in the respective comparison. Bubble size reflects log10(Fold Enrichment), with small circles log10(Fold enrichment)>0.2, medium circles 0.2 < log10(Fold enrichment)>1.3, and large circles with 1.3 < log10(Fold enrichment)>2.5. Cells without bubbles indicate that the process was not significantly enriched in that comparison (FDR < 0.05).

GO enrichment analysis was performed to validate the differential expression patterns and provide functional context for the proteomic changes. The empty vector pBY triggered a massive reprogramming of proteome, with significant enrichment of defense and oxidative stress-related responses (ex. removal of superoxidase radicals, cell wall and polysaccharide catabolism) and downregulation of photosynthesis, chloroplast associated functions, carbon assimilation and nitrogen/ammonia metabolism (Fig 7). This pattern is consistent with plant stress responses [55]. Using the pBY condition as the reference in our analysis, we compare the proteomic profile of the IL1-His and IL15-His condition, to the proteomic profile of leaves infiltrated with empty vector. We identified that there are specific enriched pathways that may be linked to the production of the cytokines. From this comparison, we observed that both recombinant protein expression systems demonstrated similar core metabolic adaptations, with enrichment of glycine metabolic processes, confirmed by upregulated expression of glycine cleavage system P protein and related enzymes. At the same time the enrichment of photosynthesis pathways was observed by comparison with empty vector control, which was validated by increased abundance of oxygen-evolving enhancer proteins, and photosystem components (Fig 7). The downregulated processes also showed similar patterns, with both expression systems suppressing carbohydrate catabolism and various metabolic processes. Within the shared framework, IL1-His specifically showed the tendency to downregulate the removal of superoxide radicals (as shown previously by downregulation of peroxidase and thioredoxin reductase), which may indicate active suppression of defense mechanisms to reallocate resources toward protein production (Fig 7). At the same time, unique pathways for IL15-His associated with enrichment of protein folding and quality control pathway, which correlated with upregulation of Hsp-70, DnaK, osmotin, calreticulin, and heterochromatin organization processes with increased histone H2A and H4. Meanwhile, downregulated pathways of IL15-His were more broadly associated with metabolic and regulatory processes rather than specific defense pathways (Fig 7). Collectively, these observations suggest that, although the major component of stress response is attributed to pBY system itself, IL1-His and IL15-His expression could superimpose additional proteomic adjustments.

### Global metabolomic changes highlight distinct responses to IL1-His and IL15-His expression

To complement the proteomic analysis and further delineate the metabolic consequences of recombinant protein expression, we performed global metabolomic profiling of N. benthamiana leaves, expressing IL1-His, IL15-His, empty vector pBY and non-infiltrated leaves. A total of 2,580 metabolites were identified and quantified across all experimental groups.

**Fig 8:**
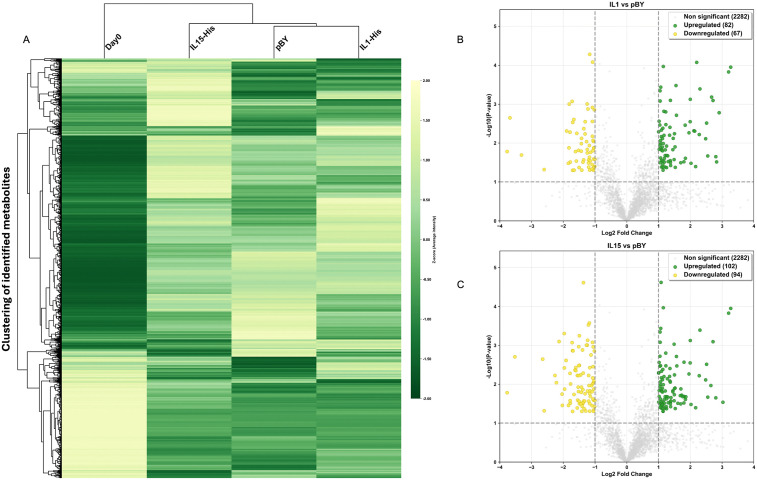
Global metabolomic profiling of *Nicotiana benthamiana* host responses to agroinfiltration and recombinant cytokine expression. (A) Hierarchical clustering and metabolite abundance. Relative abundance of 2,580 identified metabolites across four experimental conditions. Each row represents individual metabolites, and each column represents a pooled sample replicate (n = 3). Columns and rows are clustered by average-linkage hierarchical clustering on Euclidean distances computed from log2-transformed, row-wise Z-scored intensities. The colour scale indicates the row-wise Z-score, with yellow representing high relative abundance and green representing low relative abundance. (B) Volcano plot of differential metabolite abundance, showing the statistical significance and fold change distribution of metabolites altered by IL1-His, IL15-His expression in comparison with pBY empty vector. Green points are significantly upregulated, yellow points are downregulated, and gray points are non-significant proteins. Dashed lines indicate significance thresholds (p < 0.05 and |Log2FC| > 1.0). Significantly changed KEGG annotated proteins listed in S5 Table.

Hierarchical clustering of metabolomic profiles highlighted distinct metabolic signatures for each condition (Fig 8A). The non-infiltrated control leaves formed a distant cluster, establishing the baseline metabolic state. Upon infiltration with empty vector (pBY), the metabolome shifted significantly, showing the effect of the *Agrobacterium* infiltration and geminiviral vector replication activity. While the metabolic profile of IL1-His expressing leaves showed some overlap with pBY control, IL15-His expressing leaves clustered separately, indicating that IL15-His expression triggered larger shift in the metabolomic profile compared to both pBY and IL1-His.

To quantify these perturbations, we compare the metabolite abundances of the cytokines expressing leaves against the empty vector control. Volcano plot analysis (Fig 8B) indicated that IL1-His expression resulted in significant alteration of 149 metabolites (82 upregulated and 67 downregulated). In contrast, IL15-His expression induced higher total number of significantly different features, with 196 significantly altered metabolites (102 upregulated and 94 downregulated). To facilitate biological interpretation, these significantly differentially abundant features were annotated in KEGG identifiers (S5 Table). This metabolite set was used for pathway topology analysis.

### Pathway impact analysis identifies phenylpropanoid biosynthesis as a specific stress signature of IL15-His expression

**Fig 9:**
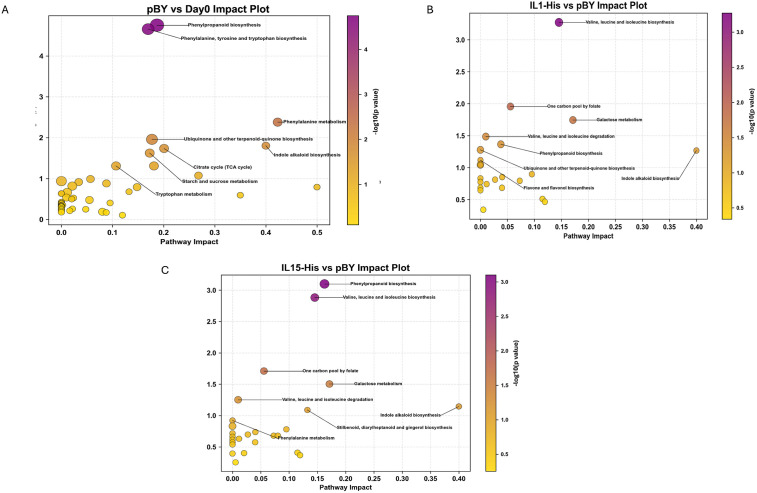
Metabolomic pathway impact analysis of *Nicotiana benthamiana* responses to agroinfiltration and cytokine Expression. (A) Impact plot of pathways altered by IL1-His expression. (B) Impact plot of pathways altered by IL15-His expression. (C) Impact plot of pathways altered by empty vector pBY. Each circle represents a single metabolic pathway. The circle's colour corresponds to its statistical significance (−log10(p-value)), and its size corresponds to its pathway impact score (based on metabolite centrality).

We mapped the annotated significant metabolites to biological pathways to identify the specific processes driving the differences in the IL1-His and IL15-His expression. Initial comparison between non-infiltrated leaves and pBY vector revealed that phenylalanine, tyrosine, and tryptophan biosynthesis were the most impacted pathways (Fig 9A). Phenylalanine and tryptophan metabolism, citrate cycle, indole alkaloid biosynthesis, starch and sucrose metabolism and ubiquinone and other terpenoid-quinone biosynthesis showed moderate enrichment (Table 2). Overall, the data indicated a significant enrichment of shikimate-derived and phenylpropanoid pathways in response to agroinfiltration itself.

**Table 2:** List of significantly impacted KEGG pathways through all comparisons.

Pathway	-log10(p)	Impact
**pBY vs Day0 comparison**		
Phenylpropanoid biosynthesis	4.75	0.187
Phenylalanine, tyrosine, and tryptophan biosynthesis	4.65	0.169
Phenylalanine metabolism	2.37	0.423
Ubiquinone and other terpenoid-quinone biosynthesis	1.95	0.177
Indole alkaloid biosynthesis	1.80	0.4
Citrate cycle (TCA cycle)	1.73	0.200
Starch and sucrose metabolism	1.62	0.172
Tryptophan metabolism	1.30	0.107
Glyoxylate and dicarboxylate metabolism	1.30	0.181
Tyrosine metabolism	1.07	0.268
**IL1-His vs pBY comparison**		
Valine, leucine, and isoleucine biosynthesis	3.27	0.14
One carbon pool by folate	1.95	0.05
Galactose metabolism	1.74	0.17
Valine, leucine, and isoleucine degradation	1.48	0.01
Phenylpropanoid biosynthesis	1.36	0.037
Ubiquinone and other terpenoid-quinone biosynthesis	1.27	0
Indole alkaloid biosynthesis	1.26	0.4
Flavone and flavonol biosynthesis	1.11	0
Glucosinolate biosynthesis	1.04	0
Phenylalanine metabolism	1.03	0
**IL15-His vs pBY comparison**		
Phenylpropanoid biosynthesis	3.09	0.16
Valine, leucine, and isoleucine biosynthesis	2.88	0.14
One carbon pool by folate	1.71	0.05
Galactose metabolism	1.50	0.17
Valine, leucine, and isoleucine degradation	1.25	0.01
Indole alkaloid biosynthesis	1.14	0.4
Stilbenoid, diarylheptanoid and gingerol biosynthesis	1.08	0.13

When comparing IL1-His expression with pBY empty vector control, the distinct metabolic profile emerged (Fig 9B). The most significantly enriched pathway with the largest impact was valine, leucine, and isoleucine biosynthesis, with concomitant enrichment of valine, leucine and isoleucine degradation, one-carbon pool by folate, and galactose metabolism (Table 2). Pathways related to phenylpropanoids (phenylpropanoid biosynthesis, ubiquinone and other terpenoid–quinone biosynthesis, flavone and flavonol biosynthesis) were also among the enriched pathways in IL1-His expressing leaves, but to a lower extent than observed in the empty vector infiltrated leaves.

For the IL15-His leaves (Fig 9C), phenylpropanoid biosynthesis again emerged as one of the most impacted pathways, together with valine, leucine, and isoleucine biosynthesis. Additional pathways with moderate impact included one-carbon pool by folate, galactose metabolism, valine, leucine and isoleucine degradation, phenylalanine metabolism, indole alkaloid biosynthesis, and stilbenoid, diarylheptanoid and gingerol biosynthesis (Table 2). As compared to pBY empty vector, IL15-His appeared to be associated with phenylpropanoid-related routes, whereas the IL1-His showed the stronger emphasis on BCAA metabolism. These results indicate that IL1-His and IL15-His expression impact distinct sets of pathways within central carbon metabolism and phenolic compounds metabolism.

In summary, IL1-His accumulated to detectable levels, whereas IL15-His remained below detection, in line with its poor yield. Across proteome and metabolome data, both cytokines largely shared the core pBY-driven response, but IL1-His appeared more compatible with host metabolism, showing modest remodeling of amino acid pathways. By contrast, IL15-His was associated with stronger induction of protein folding and stress-related processes together with enhanced phenylpropanoid-linked metabolism, suggesting that a heavier folding and phenolic-stress burden may contribute to its low accumulation, although additional limiting factors are likely involved.

## Discussion

This study investigated the differences of the plant responses, underlying differential production yields of IL1-His and IL15-His in *Nicotiana benthamiana* using *Agrobacterium*-mediated agroinfiltration. Based on our preliminary western blot data with several interleukins at 4 dpi after infiltration, IL1-His showed the strongest signal by comparison with others, while IL15- His showed no detectable accumulation. (S5 Fig). Based on this contrast, we selected IL1-His and IL15-His as representative cytokines with high and low accumulation, respectively, to compare the associated host proteomic and metabolic responses. Following the repeated agroinfiltration with the identical vector backbone, both constructs induced comparable visible necrosis at infiltration sites by 4 dpi. Nevertheless, only IL1-His accumulated to detectable levels. This contrast was confirmed by LC-MS peptide identification analysis, which identified IL1-His peptides with a 83% coverage, while no peptides for IL15-His were detected.

The difference in accumulation could be associated with several possible explanations, including disrupted folding or trafficking, reduced transcription, or translation efficiency, and/or enhanced degradation of IL15-His. In our case, both IL15-His and IL1-His were expressed from identical expression cassettes, the two genes are under the control of the same promoter and regulatory elements and would be expected to be transcribed with similar efficiency. A purely transcriptional explanation, therefore, remains possible but is unlikely to be the principal cause, and should be excluded experimentally by the investigation of the mRNA level of cytokine upon expression. More probable explanation lies downstream of translation: because both proteins carry an N-terminal signal peptide and a C-terminal ER-retention motif, the newly synthesized polypeptides would be directed into the secretory pathway, where they would be monitored by the endoplasmic reticulum quality control system. In case of incomplete or incorrect folding, the newly synthesized proteins would be targeted for disposal through ER-associated degradation or downstream proteolysis. [56]. IL15-His could be especially vulnerable to this type of post translational loss because of its complex structure. IL15 possesses a four-helix bundle architecture which, in its native context, often relies on receptor-partner stabilization of IL15-Rα [57]. By contrast, IL1-His adopts a β-trefoil compact fold. [26]. These requirements of IL15 would impose a greater folding and quality‑control burden on the plants, so that a larger fraction of the newly synthesized protein is eliminated before it can accumulate. This burden could be increased by IL15 post‑translational requirements: its mature domain carries two native disulfide bonds and three potential N‑glycosylation sites [58]. The significant increase of folding chaperones in the plants expressing IL15-His shown in our differential analysis, like calreticulin (an endoplasmic‑reticulum chaperone of the calnexin/calreticulin glycoprotein‑folding cycle) together with HSP70‑family chaperones could plausible contributing checkpoints to the post-translational loss of IL15, yet the precise quality‑control pathway affected remains to be defined. This could be possibly confirmed by the investigation of expression of the IL15-His with co-expression of the proteasome inhibitors [59]. The overall charge is another potential contributor to the different level of accumulation. Although IL15-His predicted to be negatively charged (pI = 6.8) in plant’s endoplasmic reticulum (pH approx. = 7.1) [60], and should have gained more soluble state through the electrostatic repulsion [61], these properties depend on the structural context of the individual ionizable residues. In the plant secretory pathway, the lumen acidifies from about pH 7.1 in the ER to about pH 6.3 in the trans-Golgi network/early endosome [60], which would substantially increase histidine protonation and could alter local electrostatic interactions within IL-15. By sequence inspection of the structurally characterized mature cytokine, IL-15 contains more histidine residues than mature IL-1β, making such pH-dependent local charge effects plausible. However, the existence of the SEKDEL motif prevents the transfer through the Golgi apparatus and endosome environment. Moreover, according to our screening analysis, the expression of the IL15-His without SEKDEL motif did not improve the accumulation, and IL15-His remains undetectable by our methods of analysis. It is suspected that the most plausible explanation is unfolded protein stress, which leads to protein degradation. Plant-expression data are consistent with this interpretation: human IL-15 can be produced in *N. benthamiana*, when it is fused to stabilizing partners such as Fc, while still showing lower accumulation [62].

Rather than try to do the protein-engineering of IL15-His, which would target only single protein, we sought to identify which cellular and metabolic states are associated with successful and unsuccessful protein accumulation. To explore these possibilities, we examined the proteome and metabolome profiles of the infiltrated leaves, where each cytokine was expressed, along with empty vector pBY and non-infiltrated leaves as a control. Proteomic and metabolomic measurements were performed on a single time-point at 4 dpi, which was generally optimal time showing highest recombinant protein production yield in *N. benthamiana* [63,64]. Further studies could render temporal dynamic investigations to capture more on the proteins and metabolites changed at the earlier times.

Our differential proteomic analysis revealed that Agrobacterium infiltration with an pBY vector as well as replication of viral vector upon transfer triggers a natural and conserved plant defense response. Upregulation of pathogenesis-related defense and cell-wall degradation proteins aligns with previous reports, showing that disarmed Agrobacterium strains induce defense gene expression and antimicrobial pathways [65,66]. A similar defense-associated proteomic signature is also observed in plants expressing IL1-His and IL15-His, indicating that cytokine production occurs against a background of Agrobacterium-induced stress.

Plants expressing IL1-His and IL15-His both showed an upregulation of core metabolic processes, including glycine/serine metabolism and ATP-generation process, consistent with general stress compensation. Transient production of recombinant proteins can be energetically demanding, and the observed upregulation of primary metabolic pathways reflects the attempt to meet increased ATP/NADPH need for protein folding and quality control [11]. However, only IL15-His leaves exhibited a pronounced enrichment of protein quality-control machinery, including 70 kDa heat shock cognate (Hsp-70), DnaK chaperone, endoplasmic reticulum (ER) luminal chaperone and calreticulin. The accumulation of ER-resident chaperones and heat shock proteins is commonly taken as a molecular signature of an unfolded protein response (UPR) [67–69]. These findings align with previous works, where specific complex disulfide-rich recombinant proteins induce ER stress and poor yields in plants [70]. Along with induced UPR, we found downstream stress effects in IL15-His condition, which were not observed in the IL1-His protein. The strong downregulation of CP12−1, a small chloroplast protein known to form a complex with glyceraldehyde-3-phosphate (GAPDH) and phosphoribulokinase (PRK) [71], could contribute to photosynthetic suppression.

Despite the differences in complexity of the protein structure, IL1-His and IL15-His also may place different demands on the plant protein production machinery. IL15 retained at least one predicted N-glycosylation site as reported in UniProt database, P40933, while IL1 domain lacks such motifs and is expected to remain non-glycosylated. Under these conditions, IL15-His would be more likely to go through calnexin/calreticulin lectin chaperone pathway, which selectively engages with N-glycoproteins to assist folding [72]. The specific upregulation of the calreticulin, observed in IL15-His leaves, could be consistent with an increased load of slowly folded or misfolded IL15-His. Based on assumption, we propose that IL15-His is more strongly retained in the N-glycan–dependent calnexin/calreticulin quality-control cycle and more frequently targeted for ER-associated degradation, whereas IL1-His, may bypass this checkpoint and is therefore accumulated to moderate levels. This model is hypothetical and remains to be experimentally tested. However, it offers a coherent explanation linking cytokine structure, predicted glycosylation state, and the selective upregulation of calreticulin and UPR markers. It could explain the contrasting yields of IL1-His and IL15-His.

In addition to these proteomic signatures of ER stress and protein folding activation, the untargeted metabolomic profiling provides an independent layer of information on how IL1-His and IL15-His expression affects host physiology. Our data suggests that the agroinfiltration process itself triggers a basal stress response, characterized by the activation of shikimate-derived metabolism and phenylpropanoid biosynthesis. As phenylpropanoid biosynthesis is a downstream branch of the shikimic acid pathway, these shifts likely represent a generalized response to Agrobacterium infection and viral vector delivery, both of which are known to impact the production of lignin, flavonoids, and other defense-related phenolics [73].

Building on this basal response, the results showed that IL1-His and IL15-His elicit partly distinct profiles, particularly in central carbon metabolism and phenolic compounds production. Pathway impact analysis indicated that phenylpropanoid biosynthesis was among the most strongly affected pathways specifically in IL15-His leaves, whereas IL1-His leaves display less prominent shifts in this pathway. Although these results are based on a single time point and do not allow causal inference, they are consistent with the hypothesis that IL15-His expression is associated with a more pronounced activation of specific defense-related branches of secondary metabolism. In other words, while proteomics highlights the induction of defence proteins and stress chaperones, metabolomics reveals the corresponding accumulation of defence-associated metabolites, suggesting a coordinated activation of both protein-level and metabolite-level defence responses.

This metabolic reprogramming observed in IL15-His expressing leaves could contribute to poor protein accumulation via promotion of both cell wall fortification (via enhanced phenylpropanoid and putative lignification responses) and the formation of a locally unfavourable, potentially cytotoxic environment [16,17]. In this scenario, phenolic compounds associated with phenylpropanoid biosynthesis, previously linked to cell-wall reinforcement, antimicrobial activity and reduced transformation efficiency might increase oxidative and metabolic stress, thereby predisposing IL15-His to impair folding or accelerated degradation.

In contrast, IL1-expression may appear to remain below required to fully activate this defense module. Instead, these leaves exhibited a stronger emphasis on branch-chain amino acid (BCAA) metabolism. Given that branch-chain amino acids (BCAA) biosynthesis and catabolism are important for nitrogen storage and respiratory substrates [74], the coordinated changes in these pathways could be associated with increased energy metabolism in response to IL1-His production. By avoiding the induction of a heavy phenolic-stress burden, the host cells in IL1-His-expressing leaves may maintain a more permissive environment, allowing resources to be redirected toward protein synthesis and energy production rather than defense.

Taken together, the combined proteomic and metabolomic observations are consistent with a working model in which IL15-His expression is associated with a multilayered stress response, involving protein quality-control machinery, photosynthesis-associated proteins, and activation of secondary metabolic defenses centered on phenylpropanoid biosynthesis. Beyond this data, proteomic and metabolomic profiling at multiple timepoints would elucidate dynamic of protein expression, cellular quality control mechanisms, and degradation pathways, providing crucial insights to guide optimization efforts rather than directly increasing yield. Further transcriptomic analysis could reveal genome-wide changes that fulfill proteome and metabolome data on protein accumulation and plant host responses. Implementing strategies to mitigate ER stress, such as co-expression of molecular chaperones, optimization of secretory pathway capacity, and/or modulation of secondary metabolism defense response pathways with suppressors of the shikimate pathways, may improve overall protein yield, by reducing ER stress bottlenecks. This could enhance accumulation of heterologous proteins, expressed in plants. Furthermore, the biological mechanisms uncovered in this study could be not unique to the expression of IL1-His and IL15-His. This comprehensive analytical approach could be broadly applied to improve production of other recombinant proteins, especially human cytokines in *N. benthamiana*, helping to establish plant-based systems as more efficient platforms for complex protein production.

## Conclusion

We compared the plant host responses of *N. benthamiana* upon transient expression IL1-His and IL15-His, using *Agrobacterium*-mediated agroinfiltration, integrating the observation of necrotic patterns, immunoblotting, label-free proteomics, and metabolomics profiling. Both interleukins shared identical expression strategies but exhibited different accumulation patterns. Our proteomic results indicate that IL15-His expression could be associated with unfolded protein response (UPR) and photosynthesis suppression, which could indicate the heavy folding and secretory burden on the host cells, however other factors could be involved. For IL1-His, which accumulated to detectable levels, our data showed host pathways that appear affected by general recombinant production. According to the pathway elucidated in our study, to improve the conformational stability of IL15-His and compatibility with plant machinery, the methods of reducing the folding load hold be explored, such as, e.g., stabilization mutants, fusion partners signal peptide optimization, and co-expression with ER chaperones. These approaches may provide broadly applicable solutions for enhancing the expression of other challenging recombinant proteins in plant-based systems. Furthermore, this study demonstrates the value of integrated omics as a diagnostic tool for identifying cellular bottlenecks and guiding rational optimization strategies in recombinant protein production. Collectively, these findings contribute to the development of more efficient and predictable plant expression platforms for therapeutic protein manufacturing.

## Supporting information

S1 Fig Raw non-reducing SDS-PAGE and Western blot for Figure 1. (JPG)

S2 Fig Quality control metrics and data distribution analysis of LC-MS/MS proteomics experiment. (JPG)

S3 Fig Optimization of protein filtering strategies and assessment of data quality through correlation analysis. (JPG)

S4 Fig Principal component analysis (PCA) plots of all samples (IL1-His, IL15-His, pBY and Day 0) based on their protein expression profiles. (JPG)

S5 Fig SDS-PAGE and Western blot showing expression levels of 3 interleukins–His fusion proteins (IL1-His, IL6-His and IL1-His) in *Nicotiana benthamiana.* (JPG)

S1 Table LC-MS/MS parameters for proteomic analysis. (XLSX)

S2 Table LC-MS/MS parameters for metabolomic analysis. (XLSX)

S3 Table List of high confidence identified proteins in leaf samples. (XLSX)

S4 Table List of significant proteins based on volcano plot. (XLSX)

S5 Table List of significant metabolites based on volcano plot. (XLSX)
