# Supplementary figures and images for: Comparative proteomics and metabolomics reveals distinct host protein quality control and metabolic signatures during recombinant IL1-His and IL15-His expression in Nicotiana benthamiana
Source: PLoS One. 2026 Jul 20;21(7):e0353563. doi: 10.1371/journal.pone.0353563 (PMC13384282; doi:10.1371/journal.pone.0353563)

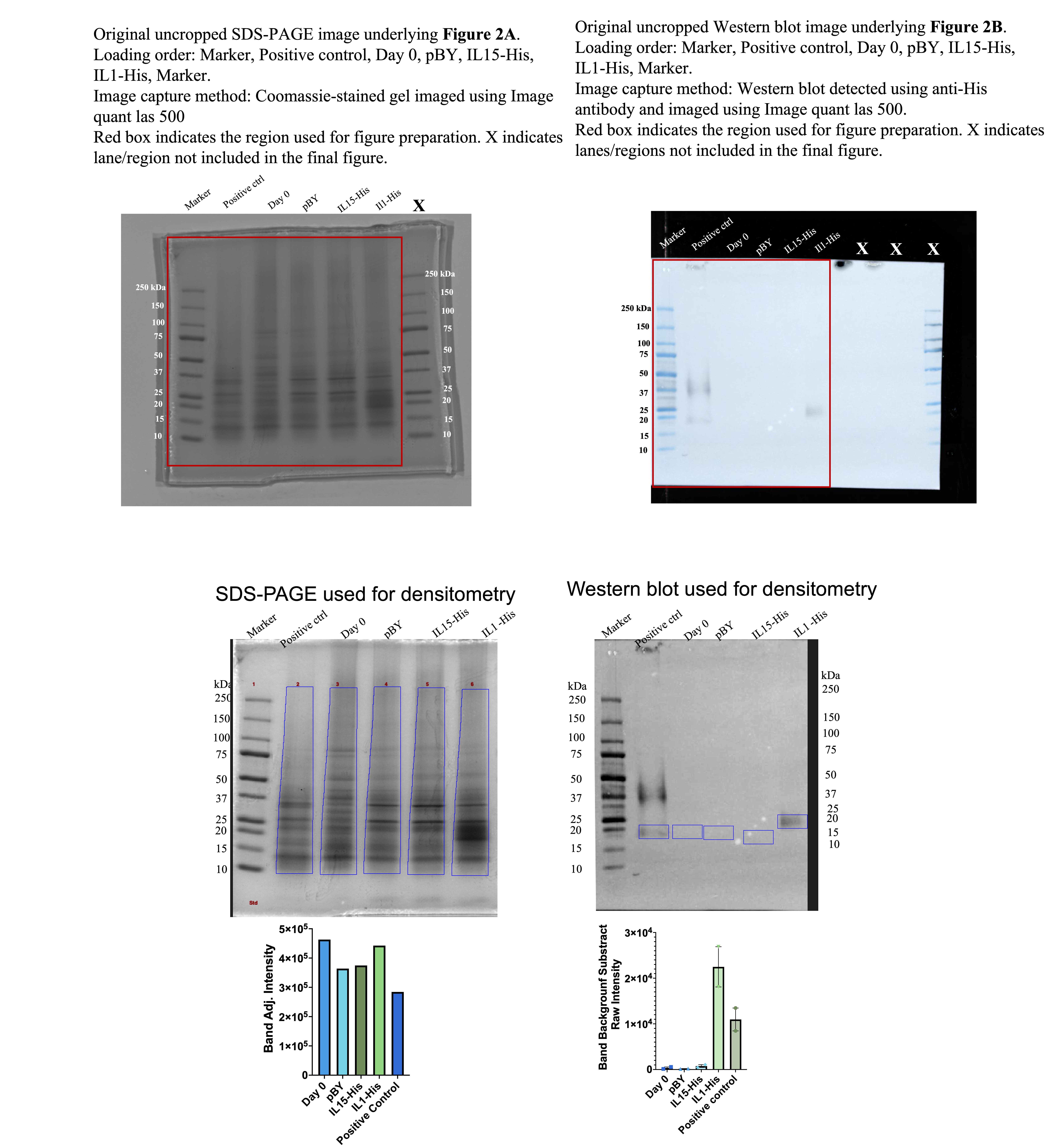

Supplement: S1 Fig — (JPG) [file pone.0353563.s001.jpg]

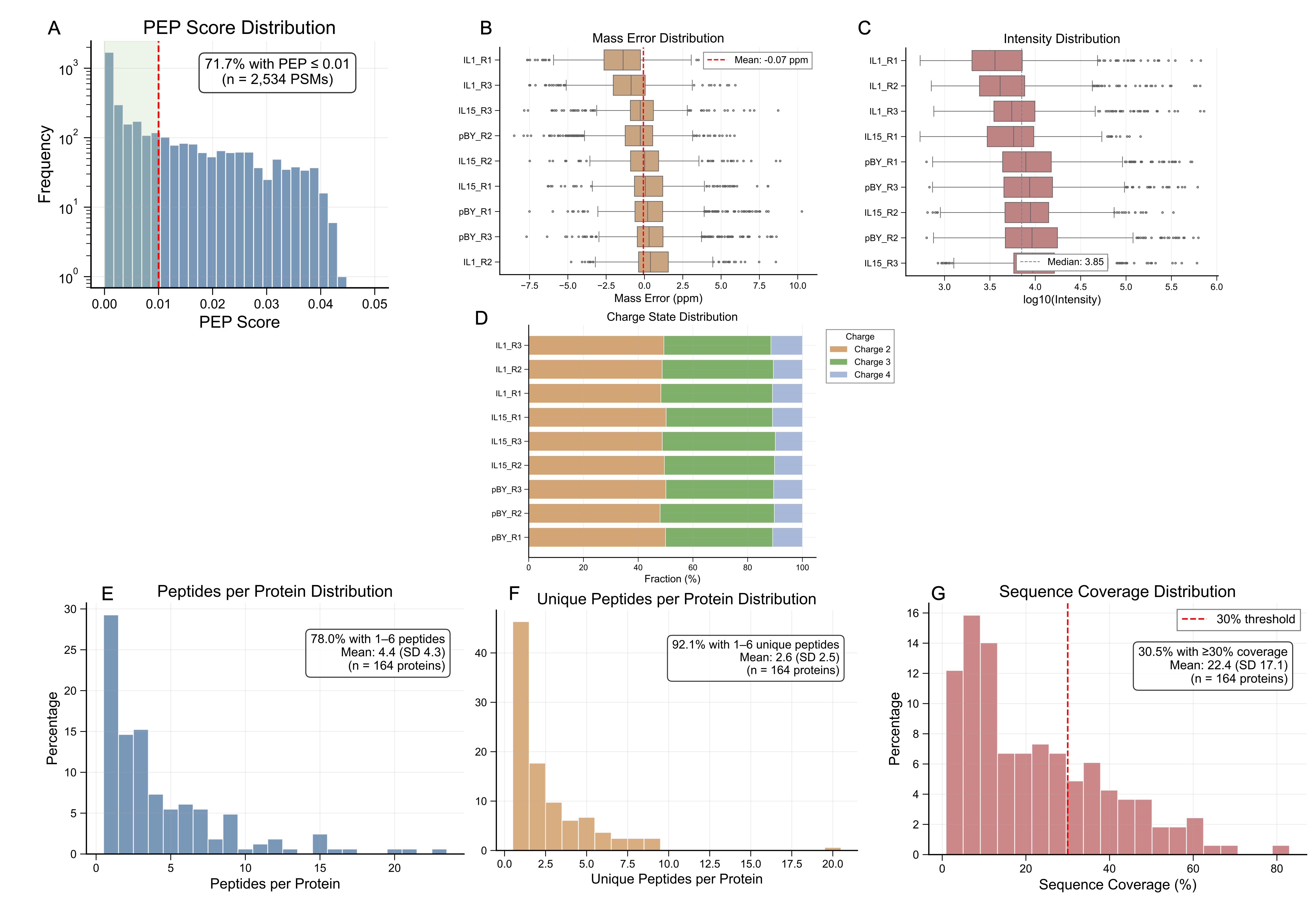

Supplement: S2 Fig — (JPG) [file pone.0353563.s002.jpg]

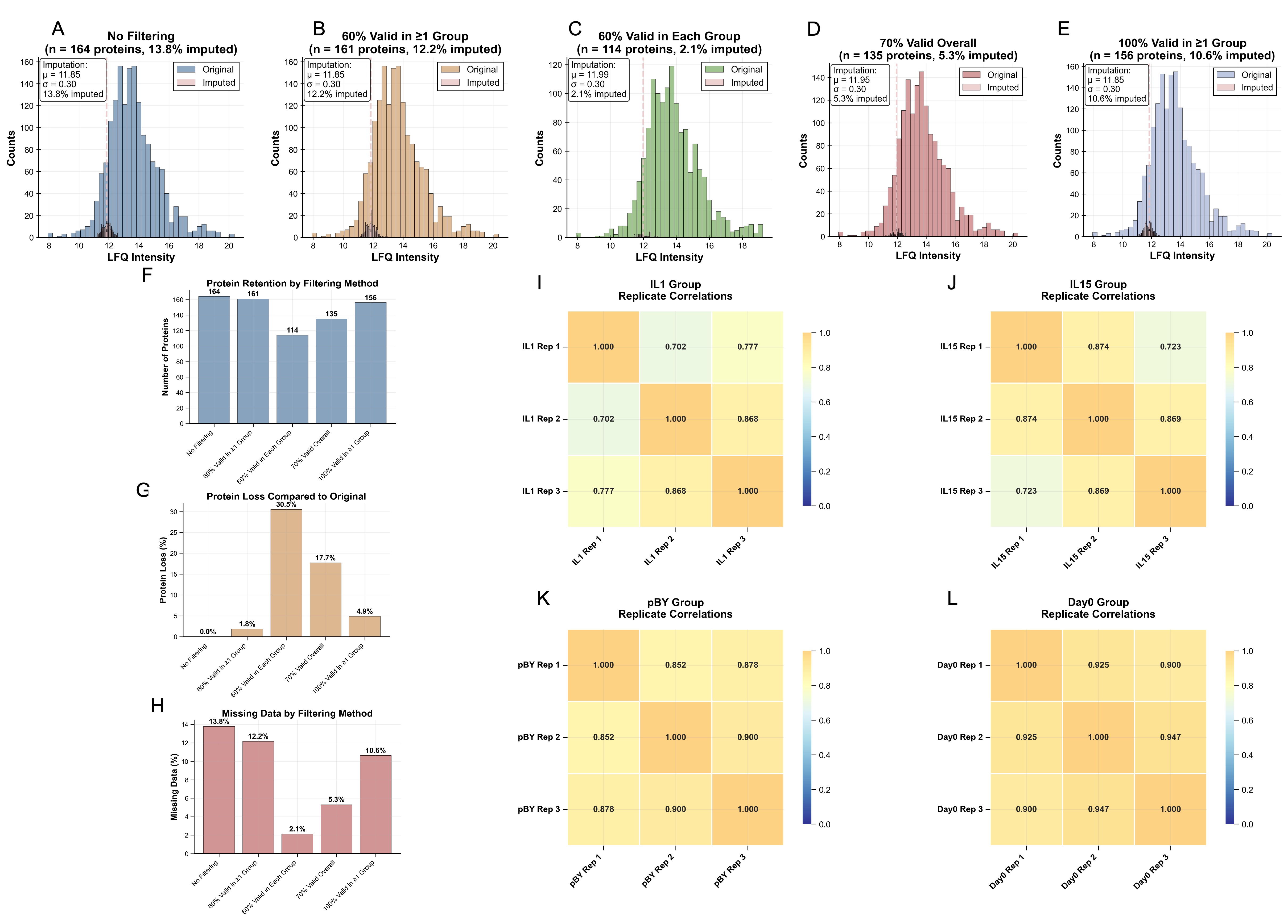

Supplement: S3 Fig — (JPG) [file pone.0353563.s003.jpg]

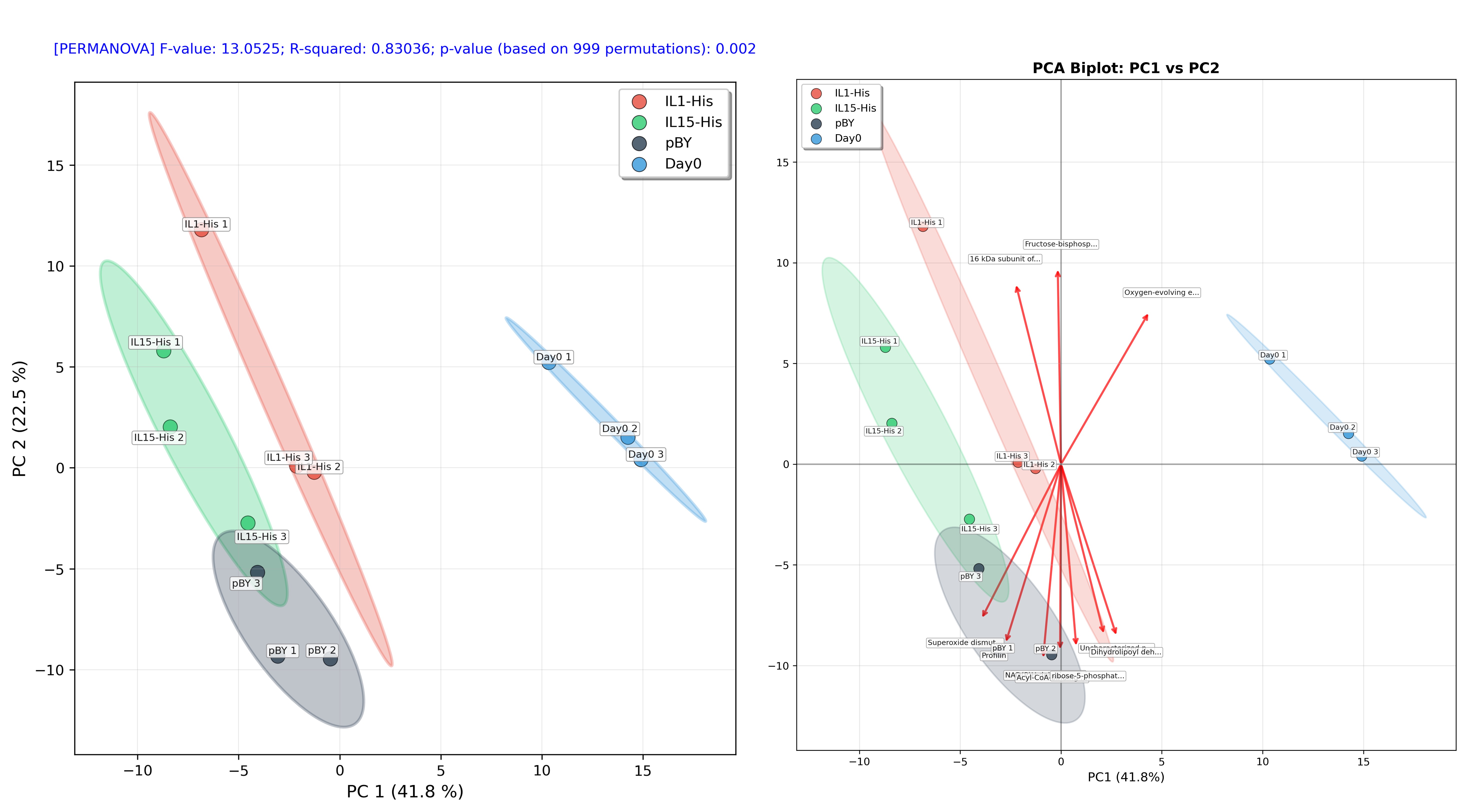

Supplement: S4 Fig — (JPG) [file pone.0353563.s004.jpg]

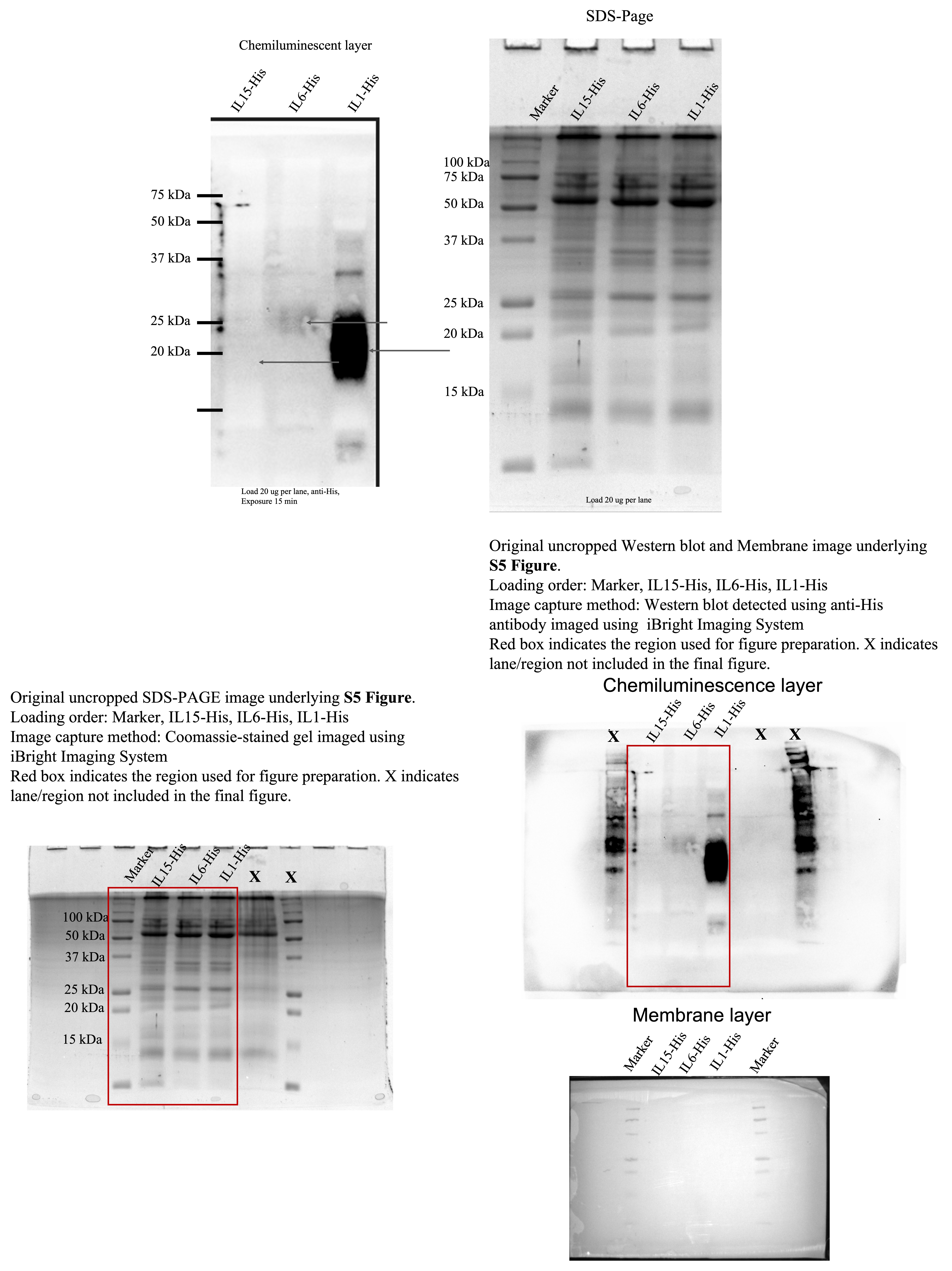

Supplement: S5 Fig — (JPG) [file pone.0353563.s005.jpg]
